# Self-acetylation at the active site of phosphoenolpyruvate carboxykinase (PCK1) controls enzyme activity

**DOI:** 10.1074/jbc.RA120.015103

**Published:** 2021-01-21

**Authors:** Pedro Latorre-Muro, Josue Baeza, Ramon Hurtado-Guerrero, Thomas Hicks, Ignacio Delso, Cristina Hernández-Ruiz, Adrian Velázquez-Campoy, Alexis J. Lawton, Jesús Angulo, John M. Denu, José A. Carrodeguas

**Affiliations:** 1 Institute of Biocomputation and Physics of Complex Systems (BIFI), University of Zaragoza, Zaragoza, Spain; 2 Wisconsin Institute for Discovery and Department of Biomolecular Chemistry, School of Medicine and Public Health–Madison, Madison, Wisconsin, USA; 3 Department of Cellular and Molecular Medicine, Copenhagen Center for Glycomics, University of Copenhagen, Copenhagen, Denmark; 4 Laboratorio de Microscopías Avanzadas (LMA), University of Zaragoza, Zaragoza, Spain; 5 Fundación ARAID, Zaragoza, Spain; 6 School of Pharmacy, University of East Anglia, Norwich, UK; 7 Instituto de Síntesis Química y Catálisis Homogénea (ISQCH), Universidad de Zaragoza, CSIC, Zaragoza, Spain; 8 Departamento de Bioquímica y Biología Molecular y Celular, Facultad de Ciencias, Universidad de Zaragoza, Zaragoza, Spain; 9 Biomedical Research Network Center in Hepatic and Digestive Diseases (CIBERehd), Madrid, Spain; 10 IIS Aragón, Zaragoza, Spain; 11 Departamento de Química Orgánica, Universidad de Sevilla, Sevilla, Spain; 12 Instituto de Investigaciones Químicas (CSIC-Universidad de Sevilla), Sevilla, Spain

**Keywords:** phosphoenolpyruvate carboxykinase (PCK1), acetylation, acetyl-CoA, enzyme inactivation, glucose metabolism, DEEP, differential epitope mapping, ITC, isothermal titration calorimetry, OAA, oxaloacetic acid, PCK1, phosphoenolpyruvate carboxykinase, PEP, phosphoenolpyruvate, STD, saturation-transfer difference

## Abstract

Acetylation is known to regulate the activity of cytosolic phosphoenolpyruvate carboxykinase (PCK1), a key enzyme in gluconeogenesis, by promoting the reverse reaction of the enzyme (converting phosphoenolpyruvate to oxaloacetate). It is also known that the histone acetyltransferase p300 can induce PCK1 acetylation in cells, but whether that is a direct or indirect function was not known. Here we initially set out to determine whether p300 can acetylate directly PCK1 *in vitro*. We report that p300 weakly acetylates PCK1, but surprisingly, using several techniques including protein crystallization, mass spectrometry, isothermal titration calorimetry, saturation-transfer difference nuclear magnetic resonance and molecular docking, we found that PCK1 is also able to acetylate itself using acetyl-CoA independently of p300. This reaction yielded an acetylated recombinant PCK1 with a 3-fold decrease in *k*_cat_ without changes in *K*_*m*_ for all substrates. Acetylation stoichiometry was determined for 14 residues, including residues lining the active site. Structural and kinetic analyses determined that site-directed acetylation of K244, located inside the active site, altered this site and rendered the enzyme inactive. In addition, we found that acetyl-CoA binding to the active site is specific and metal dependent. Our findings provide direct evidence for acetyl-CoA binding and chemical reaction with the active site of PCK1 and suggest a newly discovered regulatory mechanism of PCK1 during metabolic stress.

Protein acetylation controls a myriad of physiological processes such as gene expression (1, 2), enzyme activity (3, 4, 5, 6), or the circadian rhythm (7, 8). Although protein acetylation is facilitated by acetyltransferases, nonenzymatic acetylation has also been reported (9, 10, 11, 12). Chemical acetylation has been associated with mitochondrial proteins and is favored under alkaline pH and high acetyl-CoA concentrations (13). However, a recent report indicated that nonenzymatic acetylation also occurs on cytosolic proteins (12).

Cytosolic phosphoenolpyruvate carboxykinase (PCK1) is a rate-limiting enzyme that catalyzes the reversible conversion of oxaloacetic acid (OAA) into phosphoenolpyruvate (PEP) providing metabolites for several metabolic pathways (14). We have recently reported that PCK1 activity and stability are subject to a strong posttranslational control by acetylation, phosphorylation, and ubiquitination in response to cell energy input (15). Thus, the ability of cytosolic phosphoenolpyruvate (PCK1) to perform its reverse reaction (from PEP to OAA) depends on its acetylation level on conserved residues close to the active site (15).

Different acetyltransferases have been shown to modulate PCK1 acetylation in cells, including TIP60 (16) and p300. p300 is a histone acetyltransferase that regulates gene expression in the nucleus (17) and has been suggested to directly acetylate PCK1 in cells (18). Although we demonstrated that TIP60 was unable to acetylate PCK1 *in vitro* (15), the ability of p300 to directly acetylate PCK1 *in vitro* and the kinetic properties of the resulting acetylated enzyme were not determined. Unexpectedly, we found that purified PCK1 was only a weak substrate of p300 *in vitro* and that PCK1 alone reacted with acetyl-CoA, leading to an acetylated enzyme with significant loss of function. Here, we investigate the mechanism and molecular consequences of PCK1 self-acetylation.

By applying several biophysical and biochemical approaches, we have found that acetyl-CoA promotes the self-acetylation of PCK1, which occurs in the absence of a lysine acetyltransferase. We demonstrate that residues inside the active site, including Lys244, are efficiently acetylated, disrupting enzyme activity at low concentrations of acetyl-CoA. The crystal structure of PCK1 acetylated at Lys244 showed that acetylation of the active site impedes the coordination of substrates and forms a hydrogen bond with Arg87, preventing the motility of an essential loop. The affinity of acetyl-CoA to the active site of PCK1 depends on the presence of divalent cations. In addition, NMR studies provided a landscape of the molecular interactions between PCK1 and acetyl-CoA. Overall, our results uncover a previously unknown ability of PCK1 to undergo self-acetylation, suggesting a possible regulatory mechanism under high acetyl-CoA levels.

## Results

### Acetyl-CoA acetylates PCK1 and modifies its activity

**Figure 1:**
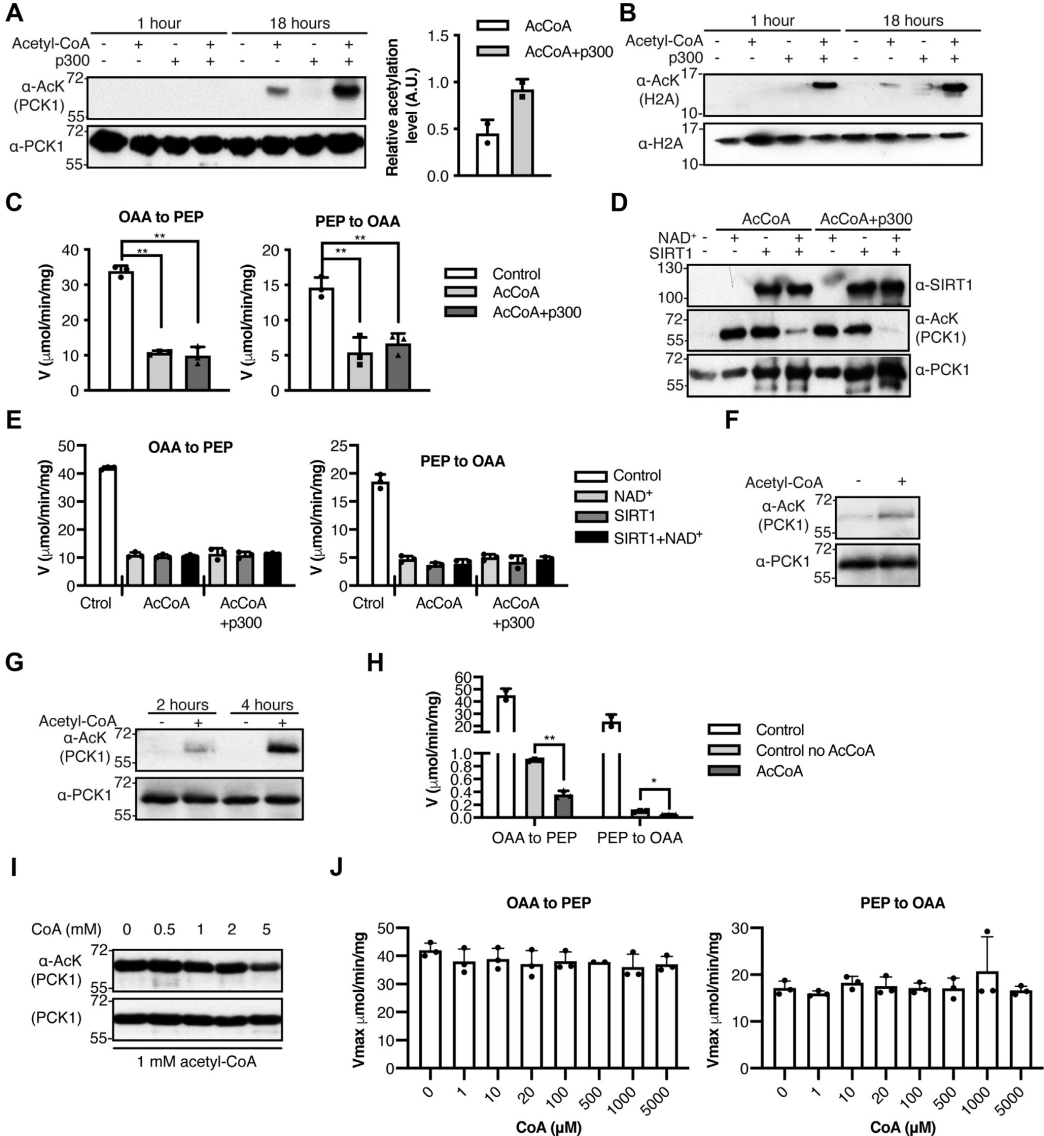
**PCK1 is chemically acetylated in the presence of acetyl-CoA.***A*, *top*, the ability of rat recombinant PCK1 to be acetylated by p300 *in vitro* was tested using different controls. However, PCK1 (α-PCK1) exhibited a high susceptibility to react and be acetylated (α-AcK) in the presence of only 1 mM acetyl-CoA, as determined by Western blotting. Numbers on the left represent molecular weight (kDa). *Bottom*, relative acetylation levels of PCK1 determined from Western blots above in the presence of acetyl-CoA or in the presence of acetyl-CoA and p300 after 18 h (n = 3). *B*, histone H2A was acetylated by p300 under the same experimental conditions described in (*A*). *C*, determination of PCK1 activity in both reaction directions for samples acetylated in the presence of acetyl-CoA *versus* acetyl-CoA+p300 obtained in (*A*) (n = 3); ∗∗*p* < 0.01. *D*–*E*, Western blot shows that SIRT1 (α-SIRT1) deacetylates (α-AcK) chemically (AcCoA) and enzymatically (AcCoA+p300) acetylated PCK1 (α-PCK1) (*D*), without recovery in enzyme activity, compared with recombinant PCK1 incubated in the absence of acetyl-CoA (*E*) (n = 3). *F*, chemical acetylation of human recombinant PCK1 after 18 h of incubation in the presence of 1 mM acetyl-CoA, as described in (*A*). *G*–*H*, similar experiment to that described for (*F*) but performed at 37 °C for 2 or 4 h (*G*). After 2 h, enzyme activity was considerably decreased, compared with the freshly thawed enzyme (control), but differences between no acetyl-CoA (control no AcCoA) and acetyl-CoA (AcCoA) treatment were observed (n = 3); ∗*p* < 0.05; ∗∗*p* < 0.01. *I*, determination of the ability of CoA to interfere with chemical acetylation of PCK1 by Western blot analysis. The acetylation reaction was carried out in the absence of CoA or in the presence of 0.5, 1, 2, or 5 mM CoA, with 1 mM acetyl-CoA in all cases. *J*, determination of PCK1 activity in both reaction directions in the presence of different concentrations of CoA. PCK1 was preincubated in the absence of CoA or in the presence of the indicated concentrations of CoA for 30 min at 30 °C and enzyme activity was assayed in buffer containing the same concentrations of CoA. PCK1, phosphoenolpyruvate carboxykinase.

The control of PCK1 activity relies on the acetylation level on certain residues (15), and p300 has been postulated as the major acetyltransferase catalyzing these events by directly binding PCK1 (18). However, the effects of direct acetylation of PCK1 by p300 on the kinetic properties of PCK1 have not been studied. To compare the kinetic properties of PCK1 between cell-mediated and *in vitro* p300-dependent acetylation, we set up experiments to acetylate recombinant rat PCK1 in the presence of recombinant human p300 and assess the level of acetylation by Western blot. After an 18-h incubation at 30 °C we observed that p300 was able to acetylate PCK1 (Fig. 1*A*). However, the controls associated with this experiment revealed that almost 25% of PCK1 was acetylated by acetyl-CoA alone when compared with PCK1 incubated in the presence of p300 and acetyl-CoA (Fig. 1*A*). In fact, p300 inefficiently acetylated PCK1 (18 h) compared with a known substrate, histone H2A (19), which was almost fully acetylated after 1 h under the same conditions (Fig. 1*B*). We also observed that PCK1 acetylated with acetyl-CoA alone or together with p300 had decreased activity (residual ∼30%) in both reactions under *V*_max_ conditions compared with controls (Fig. 1*C*). These results imply that acetyl-CoA can react with PCK1 in the absence of an acetyltransferase (hereafter, referred to as nonenzymatic or chemical acetylation).

We next sought to determine how enzyme-catalyzed and nonenzymatic acetylation affect the kinetic properties of PCK1. PCK1 was purified as a His-tagged protein and incubated at 30 °C for 18 h in the presence or absence of acetyl-CoA with or without p300. Sodium acetate (NaAc) was included as a negative control to demonstrate that a stable acetyl group in solution cannot be transferred into the PCK1 either in an enzymatic or a nonenzymatic manner as discussed below. PCK1 was repurified using His-tag affinity columns (see Materials and Methods) before performing kinetic assays. As mentioned above (Fig. 1*C*), acetylation by acetyl-CoA alone yielded a 70% to 80% reduction in *k*_cat_ for all substrates in both reaction directions, but no significant changes in *K*_*m*_ were observed, except for CO_2_, which was reduced (1.5-fold) (Table S1). This reduction in *k*_cat_ decreased the catalytic efficiency (*k*_cat_/*K*_*m*_) by a factor of 5 to 6 in the gluconeogenic reaction (OAA→PEP) and by a factor of 2 to 3 in the reverse reaction (PEP→OAA). The p300-dependent acetylation also decreased *k*_cat_ values for all substrates, and also changed *K*_*m*_ for OAA (1.8-fold increase) and PEP (5-fold decrease). This treatment decreased the catalytic efficiency (*k*_cat_/*K*_*m*_) for the substrates OAA and GTP by a factor of 7, whereas the catalytic efficiency was slightly decreased (25%) for the substrates GDP and CO_2_, and for PEP, which was increased 2-fold. These results indicate that nonenzymatic acetylation decreases overall PCK1 activity without substantial changes in *K*_*m*_ for all substrates, whereas p300-dependent acetylation changes *K*_*m*_ values for OAA and PEP favoring the reverse reaction. Changes in *K*_*m*_ for PCK1 substrates due to acetylation have been described in PCK1 purified from HEK293T cells (15). No substantial differences were observed between control and NaAc treatment, indicating that the observed results were not just due to the presence of free acetate. Together, these data suggest that enzyme-catalyzed and nonenzymatic acetylation target distinct lysine residues on PCK1.

Next, we investigated whether the observed chemical acetylation could be removed by the NAD^+^-dependent deacetylase SIRT1, which was shown to deacetylate and restore the basal kinetic properties of PCK1 (15). Although deacetylation (75%–90%) was possible (Fig. 1*D*), no activity recovery was observed (Fig. 1*E*), suggesting that those acetylated residues involved in the loss of PCK1 activity were not accessible or recognized by SIRT1.

To determine if this nonenzymatic acetylation was specific for rat PCK1, we tested whether human PCK1 ortholog (Fig. S1) was acetylated in the presence of acetyl-CoA. Under the same experimental conditions, human PCK1 was also acetylated (Fig. 1*F*). Short incubations for 2 and 4 h in the presence of acetyl-CoA were performed (Fig. 1*G*), as overnight incubation with acetyl-CoA severely neutralized human PCK1 activity. Decreased (50%) enzyme activity was observed at 2 h, with negligible activity detected after 4 h (Fig. 1*H*). Owing to the known issues of the human protein being less stable than rat PCK1, the latter enzyme was used for further experiments.

We then wondered whether CoA itself was able to inhibit PCK1 chemical acetylation or enzyme activity. First, we performed competition experiments using different concentrations of CoA (0–5 mM) in the presence of constant concentrations of acetyl-CoA (1 mM). Figure 1*I* shows that chemical acetylation of PCK1 is not prevented by the presence of CoA and only a slight inhibition is observed when using a 5-M excess of this compound. To exclude the possibility that PCK1 was inhibited by CoA, we determined PCK1 activity in both reaction directions in the presence of different CoA concentrations (Fig. 1*J*). Our results indicate that PCK1 activity is not modified by the presence of CoA, supporting chemical acetylation as the cause of altered PCK1 activity.

Collectively, these results suggest that acetyl-CoA is capable of reacting with PCK1 *via* self-acetylation, leading to a substantial decrease in PCK1 catalysis.

### Nonenzymatic acetylation dynamics in rat PCK1

**Figure 2:**
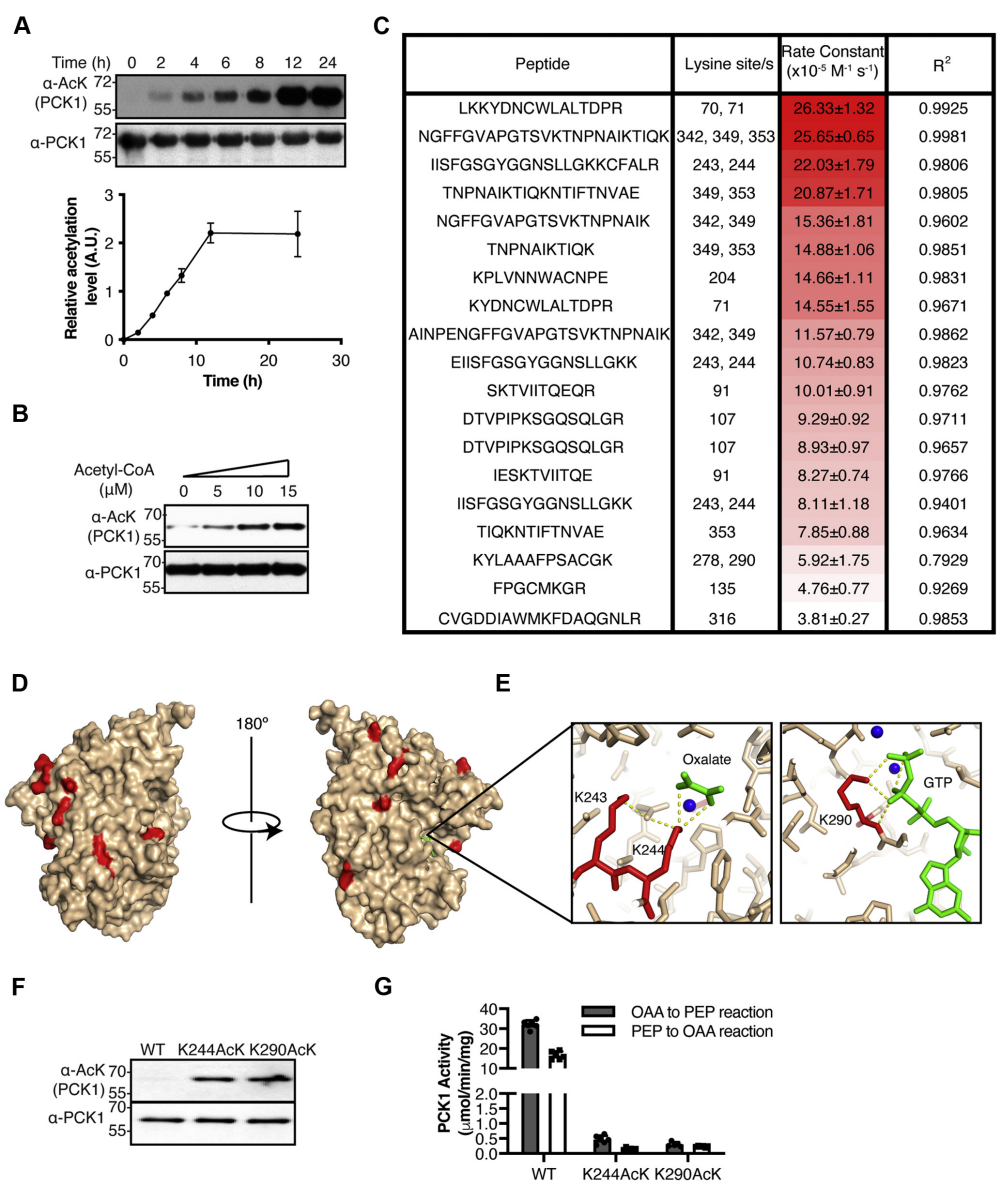
**Chemical acetylation dynamics of PCK1.***A*, *Top*, western blot analysis of chemical acetylation (α-AcK) of PCK1 (α-PCK1) along time in the presence of 1 mM acetyl-CoA. *Bottom*, graphical representation of chemical acetylation dynamics as obtained from Western blots (n = 3). *B*, recombinant rat PCK1 (α-PCK1) was treated in the presence of 1 mM acetyl-CoA and acetylation (α-AcK) was determined by Western blotting. This experiment was performed using physiological concentrations of acetyl-CoA and pH 7.4. Numbers on the left of each blot indicate the molecular weight (kDa). *C*, determination of acetylation stoichiometry (%) by MS for different residues and determination of second-order rate constants for all the quantifiable sites detected. *D*, crystal structure of rat PCK1 (PDB 3DT2) indicating nonenzymatically acetylated residues (*red*) detected in (*C*). GTP (*green*) is inside the active site. *E*, representation of the lysines (*red*) inside the active site of PCK1 detected as acetylated in (*C*). Substrates are represented in *green* and the metals coordinated to them as *blue spheres*. *F*, Western blot analysis of PCK1 K244AcK and K290AcK variants compared with WT shows acetylation (α-AcK) of these recombinant PCK1 (α-PCK1) variants purified from *E. coli* ΔCobB. Numbers on the left of each blot indicate the molecular weight (kDa). *G*, enzyme activity in both reaction directions of PCK1 K244AcK and K290AcK variants compared with WT. PCK1, phosphoenolpyruvate carboxykinase.

Incubation of PCK1 with 1 mM acetyl-CoA was sufficient to acetylate PCK1 and decrease activity by 70% in both forward and reverse reaction directions (Fig. 1). Next, we examined nonenzymatic acetylation kinetics of PCK1, revealing a linear increase in PCK1 acetylation that reached a maximum after 12 h (Fig. 2*A*).

Concentrations of acetyl-CoA in the cytosol range from 6 to 13 μM in cultured LN229 cells under high glucose conditions (20). Other reports highlight the great difference in acetyl-CoA concentrations between the cytosol (3–30 μM) and the mitochondrion (100–1500 μM) (13). However, and despite the low concentrations present in the cytosol, nonenzymatic acetylation of cytosolic proteins has been described (12). We therefore tested if nonenzymatic acetylation was possible under different low acetyl-CoA concentrations (5, 10, and 15 μM) at pH 7.4 and 30 °C for 8 h. Our results indicated that even under low acetyl-CoA concentrations PCK1 was nonenzymatically acetylated (Fig. 2*B*).

To gain insight into the kinetics of lysine reactivity for acetylation, we utilized a previously reported method using LC-MS/MS to determine site-specific second-order rate constants (21, 22). Briefly, PCK1 was incubated in the presence of acetyl-CoA to modify lysine residues as described above. Recombinant PCK1 was then denatured and chemically acetylated with D_6_-acetic anhydride, labeling all unmodified lysine residues with an isotopic acetyl group (D_3_-AcK), followed by trypsin digestion and high-resolution mass spectrometry to quantify acetylation stoichiometry. PCK1 was incubated with various concentrations of acetyl-CoA (0.5, 1, 2, 4, 8 mM) for 1 h. An aliquot of the reaction was sampled and processed for mass spectrometry to determine site-specific acetylation stoichiometry. The increase in stoichiometry of each peptide as a function of time yields a pseudo-first order rate (*k*_obs_). The second-order rate constant is determined by plotting the *k*_obs_ as a function of acetyl-CoA concentration (Fig. 2*C* and Fig. S2). Acetylation stoichiometry was determined for 30 sites (71% of total PCK1 lysines) and the second-order rate constant was determined for 14 sites, including lysines 70, 71, 91, 107, 135, 204, 243, 244, 278, 290, 316, 342, 349, and 353 (Fig. 2*C*, Table S2). The highest acetylation stoichiometry was determined for peptides containing K70/K71, K342/K349/K353, and K243/K244, with a second-order rate constant above 20 × 10^−5^ M^−1^ s^−1^ (Fig. 2*C*). Among peptides containing a single acetylated lysine, K204 and K71 exhibited the highest reactivity with second-order rate constants of 14.7 × 10^−5^ and 14.6 × 10^−5^ M^−1^ s^−1^, respectively. The lowest reactivity was detected for K316, K135, and the peptide containing lysines 278 and 290 with second-order rates of 3.8 × 10^−5^, 4.8 × 10^−5^, and 5.9 × 10^−5^ M^−1^ s^−1^, respectively.

Analysis of the crystal structure of PCK1 revealed that chemically acetylated lysines were not localized in a specific region of the enzyme (Fig. 2*D*), but interestingly, we found acetylation of two lysines inside the active site, K244 and K290 (Fig. 2*E*), with essential catalytic functions (23). To analyze the consequences of acetylation of these sites on the kinetic properties of PCK1, we used a previously described method (24) to generate site-specific acetylated PCK1 variants at positions 244 (K244AcK) and 290 (K290AcK) in *Escherichia coli* ΔCobB (22). This methodology is based on the presence of an aminoacyl-tRNA^CUA^ synthetase that incorporates site-specific acetyl-lysines at desired positions encoded by the amber codon (UAG) (Fig. 2*F*). In both cases, acetylation of either K244 or K290 resulted in an enzyme with residual activity (≤1% of controls) under saturated substrate conditions (Fig. 2*G*). In the gluconeogenic reaction (OAA→PEP), PCK1 WT, K244AcK, and K290AcK showed an activity of 32 ± 2, 0.46 ± 0.13, and 0.3 ± 0.07 μmol/min/mg, respectively, whereas in the reverse reaction (PEP→OAA) the same enzymes exhibited an activity of 17 ± 2, 0.15 ± 0.04, and 0.23 ± 0.02 μmol/min/mg, respectively. Of note, we observed that the anti-acetyl-lysine antibody used displayed different sensitivity to acetylated targets. As seen in Figure S3, the sensitivity toward K244AcK is considerably lower than for K91AcK (15), indicating that acetylation of K244 can be more extensive than suggested in Western blots (Fig. 1*A*), and therefore explaining why recovery of activity does not apparently correlate with deacetylation by SIRT1 (Fig. 1*D*). The high acetylation observed for K244 by mass spectrometry supports this view.

Together, these observations indicate that lysines in PCK1 react differently to acetyl-CoA *in vitro* and even under cell physiological acetyl-CoA concentrations. Moreover, acetyl-CoA can react with lysines from the active site, leading to PCK1 inactivation.

### Acetyl-CoA reactivity with PCK1 is specific and modulated in the presence of metals

**Figure 3:**
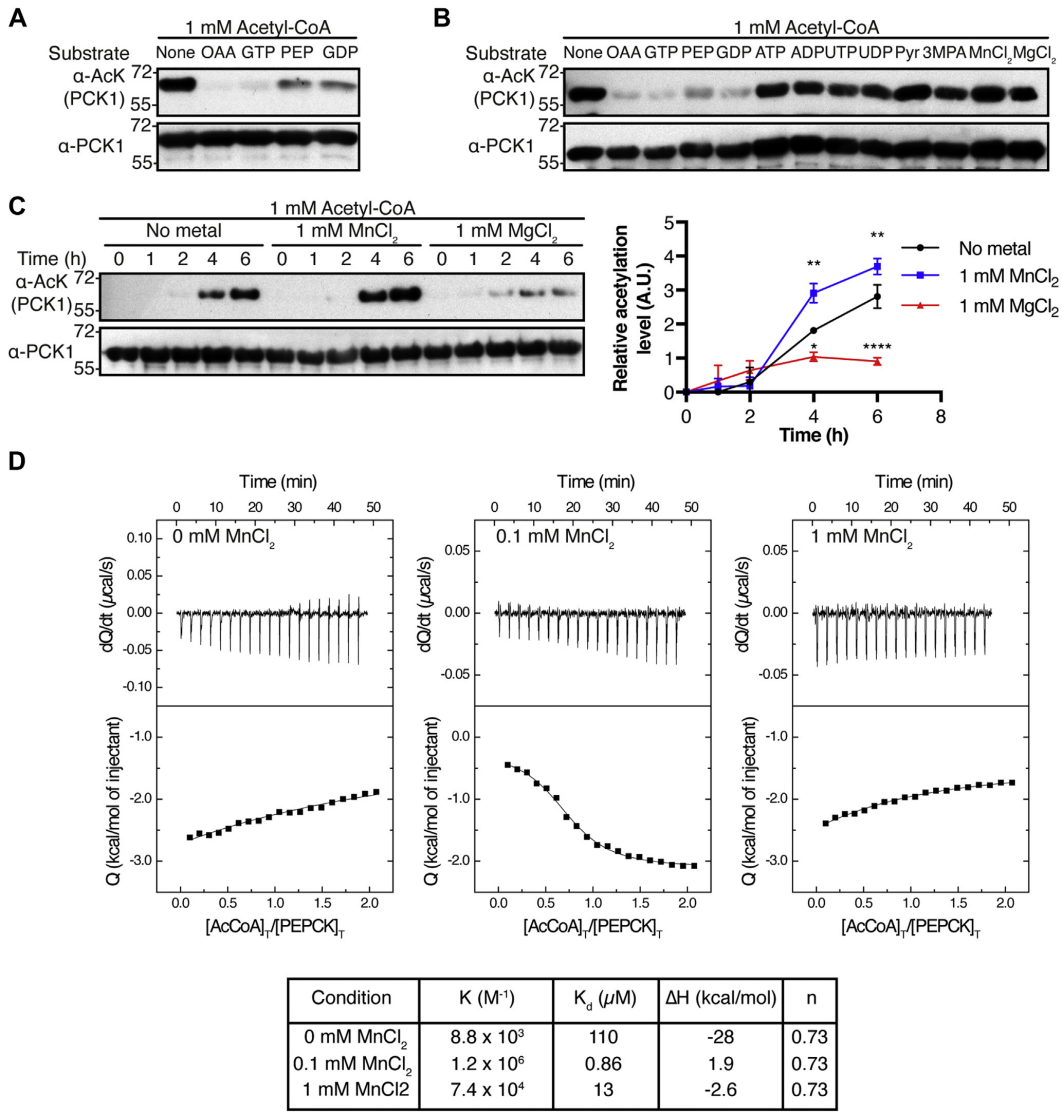
**Acetyl-CoA interaction with the active site of PCK1 is specific and metal dependent.***A*, PCK1 was incubated in the presence of only 1 mM of acetyl-CoA at 30 °C for 18 h or in the presence of other PCK1 substrates (OAA, GTP, PEP, and GDP) at a final concentration of 1 mM. Acetylation was determined by Western blotting (α-AcK) and compared with the total levels of PCK1 protein (α-PCK1). Numbers on the left indicate molecular weight. Western blot is representative of two independent experiments. *B*, similar experiment to that described for (*A*), but including other ligands to the reaction mix (ATP, ADP, UTP, UDP, pyruvate, 3-mercaptopicolinic acid, MnCl_2_, and MgCl_2_). *C*, PCK1 was incubated in the presence of 1 mM of acetyl-CoA at 30 °C supplemented with 1 mM of either MnCl_2_ or MgCl_2_. Samples were collected at different time points (0, 1, 2, 4, and 6 h), and after Western blot analysis using ImageJ, data were graphically represented (*right*). ∗*p* < 0.05; ∗∗*p* < 0.01, ∗∗∗∗*p* < 0.0001; n = 2. *D*, calorimetric titrations for the PCK1-acetyl-CoA interaction in the presence of different concentrations of MnCl_2_ at 25 °C (*left*; thermograms in upper plots, and binding isotherms in lower plots), and determination of calorimetric constants for such conditions (*right*). The relative error in the association constants (K) and the dissociation constants (K_d_) is 30%, the absolute error in interaction enthalpy (ΔH) is 0.4 kcal/mol, and the absolute error in the fraction of active or binding-competent protein (n) is 0.05. OAA, oxaloacetic acid; PCK1, phosphoenolpyruvate carboxykinase; PEP, phosphoenolpyruvate.

Given the ability of acetyl-CoA to directly react with lysines inside the active site of PCK1, we wondered whether acetylation was affected when PCK1 was incubated with 1 mM acetyl-CoA plus one of its specific substrates (OAA, GTP, PEP, or GDP) at 1 mM final concentration. Incubation in the presence of these substrates partially prevented acetylation compared with controls (incubated in the presence of only acetyl-CoA). However, each substrate prevented chemical acetylation at a different level. Substrates in the gluconeogenic reaction (OAA and GTP) prevented 90% of acetylation, whereas substrates in the reverse reaction (PEP and GDP) prevented 50% of chemical acetylation (Fig. 3*A*). To test if this inhibition was substrate specific, we repeated the experiment using similar chemicals not related to PCK1 catalysis. Apart from the aforementioned PCK1 substrates, we tested other nucleotides (ATP, ADP, UTP, and UDP), pyruvate, and 3-mercaptopicolinic acid (3-MPA, a PCK1 inhibitor). We also considered the use of metals such as Mn^2+^ and Mg^2+^ that are essential for PCK1 catalysis. Apart from PCK1 specific substrates, none of the other chemicals prevented PCK1 chemical acetylation (Fig. 3*B*) suggesting a possible competitive mechanism between acetyl-CoA and PCK1 substrates.

PCK1 catalysis relies on the presence of metals (Mn^2+^ and Mg^2+^) for the correct coordination of substrates (23). Although no differences were seen when these metals were used to prevent acetylation during overnight incubation (Fig. 3*B*), we tested if the interaction was modulated in their presence. To do so, a time-course experiment was performed incubating PCK1 in the presence of 1 mM acetyl-CoA and in the absence or in the presence of 1 mM of each metal. We observed that after 6 h the presence of MnCl_2_ enhanced PCK1 chemical acetylation by a factor of 1.3 and that the presence of MgCl_2_ decreased the acetylation rate by a factor of 3 compared with positive controls (Fig. 3*C*). In addition, isothermal titration calorimetry (ITC) assays confirmed that the presence of either 0.1 or 1 mM Mn^2+^ facilitated the interaction between PCK1 and acetyl-CoA (Fig. 3*D*). In fact, K_d_ for acetyl-CoA decreased by a factor of 100 (110 *versus* 0.9 μM) in the presence of 0.1 mM Mn^2+^ and by a factor of 9 in the presence of 1 mM Mn^2+^ (110 *versus* 13 μM). For further experiments, 0.1 mM Mn^2+^ was added to reaction mixtures. Our results indicate that the presence of Mn^2+^ mediates the interaction of acetyl-CoA and PCK1, promoting a better binding for optimal self-acetylation and enzyme inactivation.

### Acetylation of K244 by acetyl-CoA alters the active site of PCK1

**Figure 4:**
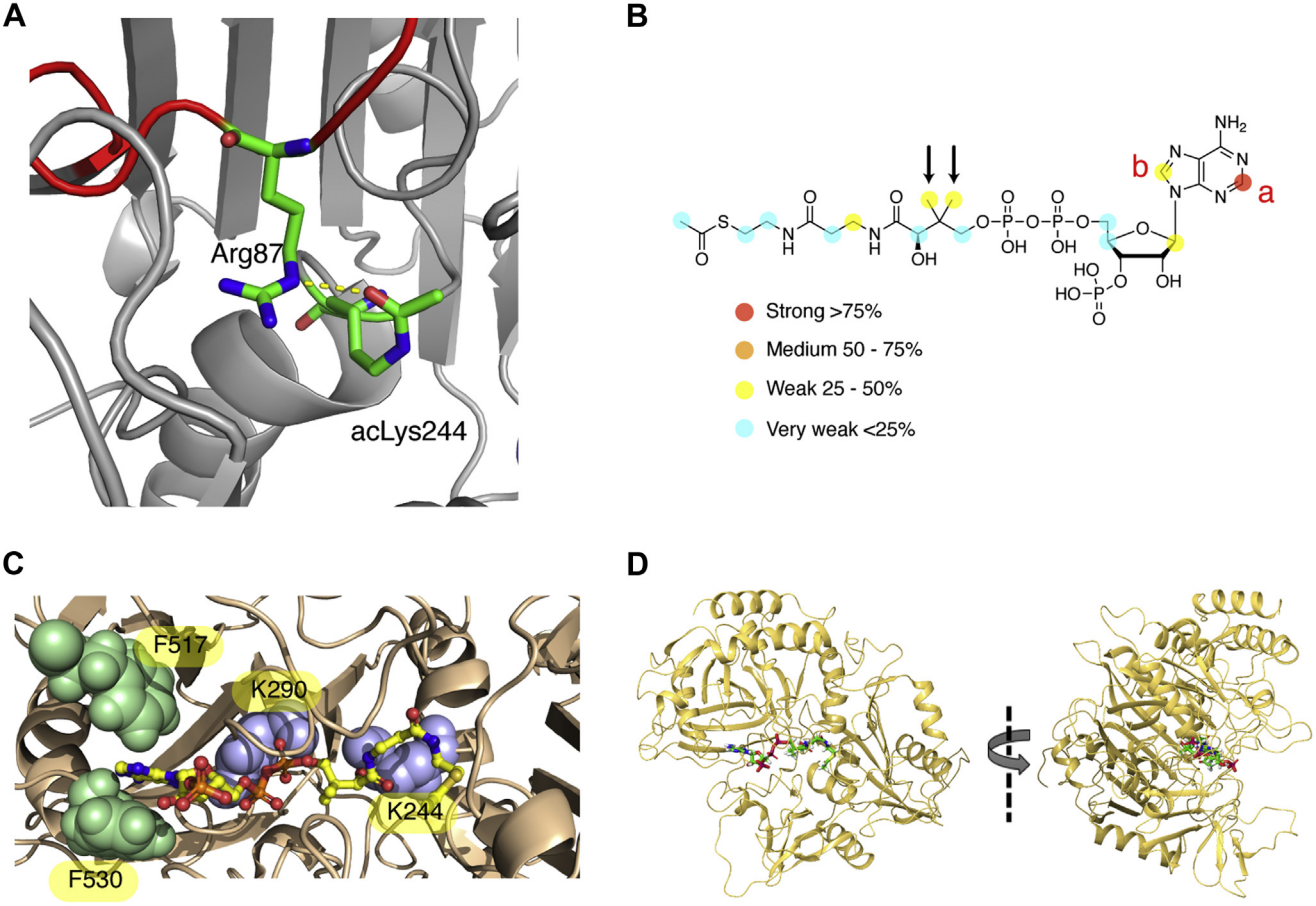
**Occupancy of acetyl-CoA inside the active site of PCK1 and determination of the effects of K244 acetylation.***A*, crystal structure of the PCK1 K244AcK mutant shows a hydrogen bond between acK244 and Arg87 that disrupts enzyme activity. The R-loop is highlighted in *red*. *B*, mapping of AcCoA-binding epitope upon interaction with PCK1 by STD NMR with saturation at −1.0 ppm. The *colored spheres* represent the normalized STD NMR intensity. Only STD responses are indicated for those protons that could accurately be measured. The letters a and b indicate the protons, and *arrows*, the methyl groups mentioned in the text. *C*, docking 3D model for the complex acetyl-CoA–PCK1. Detailed view of acetyl-CoA. The ligand is completely surrounded by PCK1 residues, in a narrow groove. Significantly, a π–πstacking involving uracyl is predicted with both F517 and F530, in agreement with STD-NMR results. *D*, full view of the docking complex, showing acetyl-CoA in the inner groove of the protein with the close side chains of K91 and K244. PCK1, phosphoenolpyruvate carboxykinase; STD, saturation-transfer difference.

To gain insight into the consequences of acetylation on K244 and the mechanism of chemical acetylation we crystalized the PCK1 K244AcK variant and PCK1 WT in the presence of acetyl-CoA. However, we were unable to obtain a complex between PCK1 and acetyl-CoA. On the contrary, we were successful in obtaining the crystal structure of PCK1 K244AcK (Table S3). The structure showed that acetylation on K244 prevents the coordination of Mn^2+^ with this residue, but interestingly the acetyl group of K244 establishes a hydrogen bond with Arg87 in the R-loop, which is essential for catalysis (23) (Fig. 4*A*). These results indicate that acetylation on K244 affects the Mn^2+^ coordination and also the interaction and synchronization of two essential loops for PCK1 catalysis, leading to PCK1 inactivation.

### Analysis of the interaction between PCK1 and acetyl-CoA by saturation-transfer difference NMR

Since crystallization of PCK1 with acetyl-CoA did not produce the expected results, in order to gain insight into the details of the interaction between acetyl-CoA and PCK1, we used saturation-transfer difference (STD) NMR spectroscopy to study the binding of acetyl-CoA and coenzyme A (CoA) to PCK1 in solution (25). STD NMR experiments showed clear STD signals for both, acetyl-CoA and CoA (see Fig. S4), confirming that they bind to PCK1 with a fast kinetics compatible with affinities in the micromolar to millimolar range, which is in agreement with our ITC experiments (Fig. 3*D*). STD NMR intensities were significantly stronger for acetyl-CoA, which, for such ligands of close structural similarity, is strongly supportive of a higher affinity of acetyl-CoA in comparison with CoA. By an analysis of STD initial growth rates, we could gain structural information about the mode of interaction of both ligands with the enzyme (26). The STD NMR-determined binding epitope mapping of acetyl-CoA in the presence of PCK1 shows that the adenosine moiety is the most important part of the ligand for molecular recognition, evidenced by the strong STD values for protons A, B, and C (Fig. 4*B*). The STD values are weaker for the rest of the molecule; interestingly, the two methyl groups of the pantothenic acid moiety stand out with slightly higher intensities (Fig. 4*B*). The pattern of STD signals in the epitope map is consistent with adenosine binding likely very close to binding site side chains with the rest of the molecule making looser contacts, supporting some degree of allowed flexibility.

The same analysis of the STD NMR experiments for CoA showed a rather comparable binding epitope map (Figs. S5 and S6), indicating that both acetyl-CoA and CoA adopt similar conformations in the state bound to PCK1, and supporting that they likely bind at the same site. To confirm this, we also ran differential epitope mapping (DEEP)-STD NMR experiments (27, 28), which provide information about the orientation of the ligand by identifying the type of protein residues making the spatial contacts. As a result, no significant differences were found for both ligands in the determined DEEP-STD factors with alternate irradiation on aliphatic and aromatic side chains of PCK1 (Fig. S7), in agreement with similar binding modes of acetyl-CoA and CoA in the same site and matching well with the homogeneous distribution of aromatic/aliphatic residues along the binding groove predicted from docking calculations (see next section). In addition, STD signals measured in the presence of Mg^2+^ did not show any difference, meaning that the ligand binding mode to PCK1 does not substantively change in the presence of the divalent cation.

These results confirm the interaction of acetyl-CoA with PCK1 identifying the atoms in the ligand involved in contacts with the protein, supporting the ability to transfer acetyl groups to reactive lysines inside the active site and indicating a higher affinity for acetyl-CoA than CoA in the active site of PCK1.

### Molecular docking 3D model of the acetyl-CoA–PCK1 complex

Starting from a previous crystallographic structure of the GTP–PCK1 complex (Protein Data Bank (PDB) ID 3DT2) and taking into account the experimentally determined acetyl-CoA-binding epitope mapping from STD NMR, a structure of the acetyl-CoA–PCK1 complex was built (Fig. 4, *C*–*D*). To that aim, the nucleotide moiety of acetyl-CoA was positioned at the same location in the binding site as GTP, whereas the pantotheine moiety was manually docked in the existing groove. The relatively small space available in the groove allowed easy docking of the pantothenic residue inside by maximizing the occupancy within the binding site. The final 3D structure of the complex was generated by consecutive steps of energy minimization and short molecular dynamics relaxation (see supporting information for further details [Fig. S8]). The resulting structure (Fig. 4, *C*–*D*) is in very good agreement with the NMR data. First, the predicted intermolecular π–π stacking interactions of the side chains of F517 and F530 with the nucleobase explain the observed enhancement of STD signal when aromatic residues are selectively saturated. Second, in the 3D docking model of the complex the ligand is completely surrounded by residues from the enzyme and no part of it is solvent exposed, in excellent agreement with the experimental binding epitope mapping of acetyl-CoA in which all hydrogens of the ligand received saturation from the protein. Finally, the proposed 3D model not only agrees with the STD NMR data but also is in excellent agreement with the ability of PCK1 to transfer the acetyl group to several lysines in this groove and the binding pocket, such as K91, K244, and K290, which are found in the proximity of the acetyl moiety. Particularly K244 is the closest to the ligand and establishes an H-bond with the hydroxyl group on the pantotheine moiety, whereas K290 is involved in a strong interaction with one phosphate unit, and is a bit farther from the ligand, which is in agreement with K290 displaying a lower second-order rate constant.

These results agree with all previous data and indicate that acetyl-CoA makes specific contacts with protein residues allowing its positioning inside the active site in a binding mode compatible with the transfer of the acetyl group to at least K244 and K290, in terms of ligand proximity.

## Discussion

Here, we have studied the ability of PCK1 to be acetylated in the presence of different concentrations of acetyl-CoA. Nonenzymatic acetylation of PCK1 results in a decreased *k*_cat_ for all substrates without changes in *K*_*m*_. Mass spectrometry experiments revealed that nonenzymatic acetylation occurs with high reactivity inside the active site of PCK1, residues K244 and K290. The interaction between acetyl-CoA and PCK1 is metal dependent, as determined by ITC experiments, and is likely a prerequisite for an optimal self-acetylation of PCK1 that leads to its inactivation. Furthermore, crystal structure of the K244AcK variant revealed that acetylation would prevent metal coordination likely impeding PCK1 binding to OAA or PEP. In addition, the hydrogen bond between the acetyl group and R87, which is involved in catalysis, would impede turnover. STD NMR experiments identified contact points in acetyl-CoA with PCK1, and DEEP-STD NMR experiments supported binding along the groove in the GTP/GDP binding site of PCK1. Molecular docking placed acetyl-CoA in the active site in a position compatible with the transfer of the acetyl group to lysines 244 and 290. Altogether, the unexpected interaction of PCK1 with acetyl-CoA inside the active site reveals two ways of controlling enzyme activity: on one side, competing with substrates for access to the active site, and, on the other, by self-acetylation of essential catalytic residues inside the active site.

Acetyl-CoA is generated in mitochondria in response to energy input and acts as metabolic sensor in the cell through protein acetylation (29). Dietary changes modify acetyl-CoA and protein acetylation levels in the cell (30, 31, 32). Acetylation of PCK1 was first discovered in yeast (16) and several acetylation sites have been reported in different mammalian species (6, 33, 34, 35). PCK1 acetylation is mediated by p300 (15) and a direct interaction between PCK1 and p300 has been reported (18). However, *in vitro* experiments performed using recombinant p300 reveal that PCK1 is a weak substrate for p300 (Fig. 1, *A*–*B*). Unexpectedly during our experiments, we observed high acetylation of PCK1 when the enzyme was incubated in the presence of only acetyl-CoA (Figs. 1*A* and 2*A*) resulting in low enzymatic activity (Table S1).

Nonenzymatic acetylation was first discovered in histones (10). Owing to the alkaline pH and high acetyl-CoA concentrations this phenomenon is highly feasible in mitochondria (9, 13, 21, 36). Several lysine acetyltransferases are regulated by autoacetylation (9, 37, 38, 39, 40, 41), although this process is also possible in proteins without known acetyltransferase activity (42). This is also the case of PCK1, which is able to directly interact with acetyl-CoA at the active site and transfer acetyl groups to adjacent lysines (Figs. 2*C* and 4, *B*–*D*). A previous work reported high reactivity of cytosolic proteins for acetylation (12), which is consistent with the ability of PCK1 to be acetylated even under the low cytosolic concentrations of acetyl-CoA (20) (Fig. 2*B*).

Acetylation modifies PCK1 activity and interconverts the enzyme from gluconeogenic to anaplerotic functions (15). However, self-acetylation at the active site, where K243 and K244 are the most reactive residues, abolishes PCK1 activity (Figs. 1 and 2 and Table S1), suggesting a different regulatory mechanism. During catalysis, K244 is essential for the coordination of the manganese directly bound to OAA (23). According to this, site-specific acetylation of K244 prevented manganese coordination and established a hydrogen bond with another catalytic residue, R87 (Fig. 4*A*), which explained the low activity observed for the enzyme (Table S1). Although PCK1 activity relies on the presence of both manganese and magnesium (23), manganese is more relevant for enzyme activity (43), being required for the coordination of acetyl-CoA to the active site (Fig. 3, *C*–*D*). Moreover, as our ITC and STD NMR studies show, acetyl-CoA appears to be correctly positioned in the active site by interaction with specific residues in that cavity, in a similar fashion as PCK1 substrates do. The binding of acetyl-CoA to the active site is metal dependent (Fig. 3) and relies on the GTP/GDP-binding site (Fig. 4) of the enzyme (44, 45), which is evident from NMR data suggesting interactions with residues F517 and F530. The binding of acetyl-CoA to PCK1 is also specific (Figs. 3, *C*–*D* and 4, *B*–*C*) and unique for a non-acetyltransferase cytosolic enzyme. This is evident from the inability of compounds others than PCK1 substrates to prevent chemical acetylation (Fig. 3, *A*–*B*), even when incubating with ATP and ADP, known substrates of bacterial (23) and yeast (16) Pck1 that share certain structural similarity to acetyl-CoA. Of interest, we found that PEP and GDP, substrates for the anaplerotic reaction of PCK1, were less efficient to inhibit self-acetylation of the enzyme (Fig. 3, *A*–*B*), compared with OAA or GTP. This may suggest a regulatory shut down mechanism under high glucose conditions (15) to decrease the supply of metabolites to the tricarboxylic acid cycle and to prevent carbon stress in the cell (36). Although the role of nonenzymatic acetylation/autoacetylation of enzymes *in vivo* must be deeply explored, here we provide detailed biochemical and structural information on the consequences of autoacetylation on PCK1, a key metabolic enzyme.

In summary, our unexpected results demonstrate that PCK1 binds acetyl-CoA and can react with sufficient kinetics and stoichiometry to significantly alter its catalytic activity. It is possible that, under cell physiological conditions with high concentrations of acetyl-CoA and low PCK1 substrates, a pool of PCK1 molecules are irreversibly inactivated by self-acetylation. However, enzyme-catalyzed reversible acetylation might be favored when acetyl-CoA and PCK1 substrates are high, allowing regulation of the directionality of the reaction, as we have described previously (15). Because of the described competition among acetyl-CoA and common substrates of PCK1 to enter the active site, PCK1 can function as a sensor of the relative amounts of those substrates and acetyl-CoA, responding with either irreversible inactivation or with a reversible modification that determines the direction of the reaction depending on the metabolic situation.

## Experimental procedures

### Purification of rat and human PCK1 from *E. coli* and kinetic assays

The human PCK1 gene was obtained by PCR using a mix of kidney, skeletal muscle, and liver cDNAs (multiple tissue cDNA panels I and II from Clontech) and cloned initially into pET15b-PP (PreScission Protease) using NdeI and SalI restriction sites. After sequencing, we identified some polymorphisms that were corrected by site-directed mutagenesis to match the consensus human PCK1 sequence (NP_002582.3). Then the cDNA was cloned into a previously reported pET22b-SUMO plasmid (46) using BamHI and SalI as restriction enzymes. Rat and human PCK1 were overexpressed and purified as described from *E. coli* BL21 DE3 Star (15). Rat PCK1 K244AcK and K290AcK constructs were generated using the Q5-site directed mutagenesis kit (New England Biolabs) and the following primers: K244AcK-Forward 5′-GCTTGGGAAGTAGTGCTTTGCGC-3′, K244AcK-Reverse: 5′-AGTGAGTTCCCACCGTATC-3′, K290AcK-Forward: 5′-TGCCTGTGGGTAGACCAACCTGGC-3′ and K290AcK-Reverse: 5′-CTGGGGAAGGCTGCTGCC-3′. *E. coli* BL21 ΔCobB cells (22) were cotransformed using either the K244AcK or K290AcK construct and an acetyl-lysine tRNA^CUA^ synthetase construct (24). Both the acetylated variants were overexpressed and purified as described (15). PCK1 kinetic assays were performed as reported (15). To determine PCK1 activity in the presence of CoA, PCK1 was incubated with increasing concentrations of CoA for 30 min at 30 °C before initiating the kinetic assay. Activity was measured in the reaction mix containing the same concentration of CoA used during incubation.

### Chemical acetylation and p300 acetylation assays

Acetyl-CoA, CoA, OAA, GTP, GDP, pyruvate, ATP, ADP, and IAA were from Sigma. PEP was purchased from Bachem. UTP and UDP were from Carbosynth. For chemical acetylation, PCK1 (6 μM) was incubated in the presence of 1 mM acetyl-CoA in a thermostatic bath at 30 °C in acetylation buffer (50 mM Hepes, pH 8, 10% glycerol, 5 mM nicotinamide, and 1 mM tris(2-carboxyethyl)phosphine) for 8 h. When specified, low acetyl-CoA concentrations (0, 5, 10, and 15 μM) and pH 7.4 were used. Time-course experiments were performed under the same conditions but collecting samples at different time points (0, 1, 2, 4, 6, and 8 h). When indicated, other substrates and compounds were added to the reaction mix. The p300 acetyltransferase assays were performed by incubating either PCK1 or Histone H2A (M2502S, New England Biolabs) (both at 6 μM concentration) in the presence of p300 (6 nM) (SRP2079, Sigma) in acetylation buffer at 30 °C for 8 h in a thermostatic bath.

In experiments involving enzyme kinetics, after chemical or p300 treatment, PCK1 was washed three times with 25 mM Hepes, pH 7.4, 500 mM NaCl, 20 mM imidazole, and 1 mM 2-mercaptoethanol and loaded into a HiTrap TALON column. Protein was eluted in the same buffer but containing 150 mM NaCl and 400 mM imidazole (15). The SUMO tag was removed using ULP1 (1:100, ULP1:PCK1 ratio) overnight in the cold. Protein was loaded again into a HiTrap TALON column equilibrated in 25 mM Hepes, pH 7.4, 300 mM NaCl, 20 mM imidazole, and 1 mM 2-mercaptoethanol and collected in the flow-through. PCK1 was flash-frozen and stored in 25 mM Hepes, pH 7.4, and 1 mM tris(2-carboxyethyl)phosphine at −80 °C.

After each experiment, SDS-PAGE loading buffer was added, samples were boiled, loaded (1 μg per well) into a 10% polyacrylamide gel, and transferred onto polyvinylidene difluoride membranes, which were probed for acetyl-lysine (1:1000, 9814, Cell Signal Technology) and reprobed for PCK1 (1:2000, sc-74825, Santa Cruz Biotechnology). For Histone H2A acetylation experiments, nitrocellulose membranes were used. For kinetic assays, protein was diluted 10-fold in 25 mM Hepes, pH 7.5, and 10 mM DTT. When graphically represented, Western blots were analyzed using ImageJ (NIH) and normalized to the content of total PCK1.

### SIRT1 deacetylation assays

SIRT1 was purified as described (15). Chemically or p300 acetylated PCK1 was incubated diluted in deacetylation buffer (15) at a concentration of 1.4 μM and incubated in the presence of SIRT1 (0.35 μM) for 2 h at 30 °C.

### LC-MS/MS

PCK1 (1 mg/ml) was incubated in a total volume of 30 μl in the presence of different concentrations of acetyl-CoA (0.5, 1, 2, 4, and 8 mM) in acetylation buffer at 37 °C and 1000 rpm for 1 h using a Thermomixer. MS analysis was performed essentially as previously described (15), using the following parameters: ProteoWizard: 3.0.7374, MSConvert, was used as peaklist-generating software; the search engine was Mascot, version 2.5; the database searched was Uniprot Swissprot (Reviewed), *Rattus norvegicus*, release 2016 (8110 entries); trypsin and endoproteinase -Glu-C from *Staphylococcus aureus* V8 were used to generate peptides (trypsin cleaves peptides on the C-terminal side of lysine and arginine amino acid residues, unless a proline residue is on the carboxyl side of the cleavage site, and with a slower hydrolysis rate when an acidic residue is on either side of the cleavage site. Endoproteinase-Glu-C cleaves peptide bonds on the carboxyl side of either aspartic or glutamic acids); two missed cleavages and no nonspecific cleavages were permitted; cysteine carbamylation was considered as fixed modification and, as variable modifications, methionine oxidation, lysine acetylation, and lysine acetylation (D3); mass tolerance for precursor ions was ±10 ppm, and for fragment ions was ±0.01 Da; 1% false discovery rate was used as the score/expectation value for accepting individual spectra.

### ITC experiments

ITC experiments were performed in a MicroCal Auto-iTC200 (MicroCal, Malvern-Panalytical) at 25 °C. PCK1 and ligand solutions were prepared at 10 and 100 μM, respectively, in acetylation buffer supplemented with Mn^2+^ when indicated in the main text. In addition, ternary titrations of a ligand into PCK1 complex with a premix secondary ligand were performed in order to assess potential cooperative ligand interactions in PCK1. A stirring speed of 750 rpm and 2-μl injections were programmed, with consecutive injections separated by 150 s to allow the calorimetric signal (thermal power) to return to baseline. Experimental data were analyzed with a model for a single ligand-binding site implemented in Origin 7.0 (OriginLab, Massachusetts), allowing a direct estimation of the binding affinity and enthalpy for the interaction of PCK1 and its substrates.

### Crystallization

PCK1 K244AcK variant crystals were obtained by the sitting drop method by mixing 0.5 μl of 10 mg/ml PCK1 K244AcK variant and 0.5 μl of crystallization solution 20% PEG 5000, 200 mM sodium sulfate, and 100 mM Hepes, pH 7.5. The obtained crystals were cryoprotected in the crystallization solution described above containing 30% ethylene glycol and immediately frozen in a nitrogen gas stream cooled to 100 K.

### Structure determination and refinement

Diffraction data were collected on synchrotron beamline XALOC of ALBA (Barcelona, Spain) at a wavelength of 0.97 Å and a temperature of 100 K. Data were processed and scaled using XDS (47) and CCP4 (48, 49) software packages. The crystal structure was solved by molecular replacement with Phaser (48, 49) using the PDB entry 2QF2 as the template. The initial phases were further improved by cycles of manual model building in Coot (50) and refinement with REFMAC5 (51). Further rounds of Coot and refinement with REFMAC5 were performed to obtain the final structure. The final model was validated with PROCHECK; model statistics are given in Table S3. The asymmetric unit of the P2_1_2_1_2_1_ crystal contained one molecule of PCK1-K244AcK. The Ramachandran plot for the PCK1-K244AcK shows that 90.4%, 9.0%, 0.4%, and 0.2% of the amino acids are in most favored, allowed, generously and disallowed regions, respectively.

### ^1^H STD NMR experiments

All STD NMR experiments were performed in Tris-D_11_ D_2_O buffer at 25 mM, ß-mercaptoethanol 2 mM, pH 7.5. The protein concentration was 40 μM, whereas the corresponding ligand concentration (acetyl-CoA or CoA) was 1 mM; MgCl_2_ concentration was 40 mM, when present. STD NMR spectra were acquired on a Bruker Avance 500.13 MHz at 283 K. The on- and off-resonance spectra were acquired using a train of 50-ms Gaussian selective saturation pulses using a variable saturation time from 0.5 to 5 s, and a relaxation delay (D1) of 4 s. The residual protein resonances were filtered using a T_1ρ_-filter of 80 ms. All the spectra were acquired with a spectral width of 8 kHz and 24K data points using 512 scans. The on-resonance spectra were acquired by saturating at −1 ppm (aliphatic hydrogens) or 7.19 ppm (aromatic hydrogens), whereas the off-resonance spectra were acquired by saturating at 40 ppm. To get accurate structural information from the STD NMR data and in order to minimize the T_1_ relaxation bias, the STD build-up curves were fitted to the equation STD(t_sat_) = STD_max_∗(1-exp(-k_sat_∗t_sat_)) calculating the initial growth rate STD_0_ factor as STD_max_∗k_sat_ = STD_0_ and then normalizing all of them to the highest value.

### Docking and molecular dynamics calculations

The Schrodinger’s Maestro 2019-1 suite was used to manually dock acetyl-CoA into PCK1, employing the crystal structure of the complex PCK1–GTP as a template (PDB id 3DT2). First, the complex was prepared using the Protein Preparation Wizard tool, and water molecules and ions were removed. The protonation state for each residue was calculated with Epik for pH 7.5. GTP was replaced by adenosine diphosphate, and pantothenic acid and acetylcysteine moieties were manually added by maximizing the occupation of the space available within the groove. Next, coarse minimization (Maestro) was performed to relax potential atom clashes.

Protein coordinates were exported to a PDB file while the ligand was exported to a MOL2 file. Then, Amber16 and AmberTools18 were used for molecular dynamics, input file preparation, and trajectory processing. Protein was converted to the Amber format using pdb4amber, and the ligand MOL2 file was processed with Antechamber. LEaP was used to merge protein and ligand, the total charge was compensated with 10 Na^+^ ions, and the complex was placed in a 100-Å cubic box of TIP3P water molecules. The system was then minimized for 100.000 cycles, heated to 300 K, and equilibrated for 200 ps. Finally, 5-ns MD simulation was run, which showed no significant RMS deviations, supporting the dynamic stability of the 3D model of the acetyl-CoA–PCK1 complex.

## Data availability

All data are contained within the article. Besides, the PDB code for our structure has also been deposited at the PDB server (see the link https://www.rcsb.org/structure/unreleased/6YI9).

The mass spectrometry raw files and the result files used in this study have been deposited to the ProteomeXchange Consortium *via* the MassIVE partner repository and can be accessed through either the ProteomExchange dataset identifier PXD021745 or MassIVE ID MSV000086210. URL: ftp://massive.ucsd.edu/MSV000086210/; MassIVE ID: MSV000086210; ProteomeXchange ID: PXD021745.

## Conflict of interest

The authors declare that they have no conflicts of interest with the contents of this article.

## Supporting information

Supplementary Figures and Tables

JBC_JBC-2020-015103_11
