# Supplementary material for: Self-acetylation at the active site of phosphoenolpyruvate carboxykinase (PCK1) controls enzyme activity
Source: J Biol Chem. 2021 Jan 21;296:100205. doi: 10.1074/jbc.RA120.015103 (PMC7948413; doi:10.1074/jbc.RA120.015103)
Supplement: Supplementary Figures and Tables [file mmc1.pdf]

SUPPORTING INFORMATION

Self-acetylation at the active site of phosphoenolpyruvate carboxykinase (PCK1) controls enzyme activity

Pedro Latorre-Muro[1†*], Josue Baeza[2], Ramon Hurtado-Guerrero[1,3,4,5], Thomas Hicks[6], Ignacio Delso[7], Cristina Hernández-Ruiz[1], Adrian Velázquez-Campoy[1,5,8,9,10], Alexis J. Lawton[2], Jesús Angulo[6,11,12], John M. Denu[2] and José A. Carrodeguas[1,8,10*]

Material included:

- Figures S1 to S8.
- Tables S1 to S3. Supplemental information to Table S2 included in additional spreadsheet.

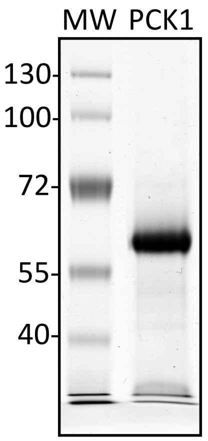

Figure S1. 10% SDS-PAGE gel of human PCK1 purified from E. coli. Numbers on the left refer to MW of markers run alongside, in kDa.

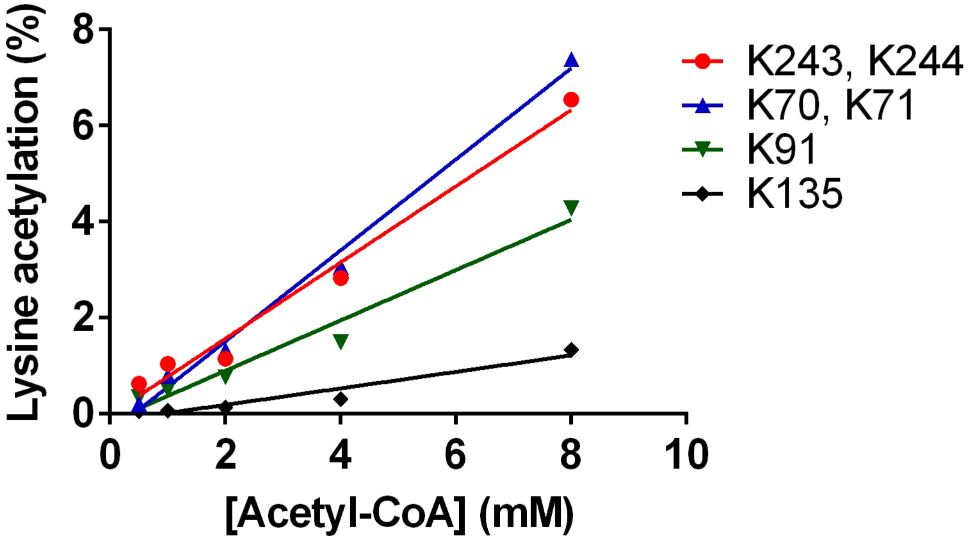

Figure S2. PCK1 chemical acetylation under different concentrations of acetyl-CoA. PCK1 was incubated in the presence of acetyl-CoA (0.5 to 8 mM). Acetylation on different residues was detected by MS and stoichiometry (%) calculated. As can be seen, each residue had different susceptibility to react with acetyl-CoA.

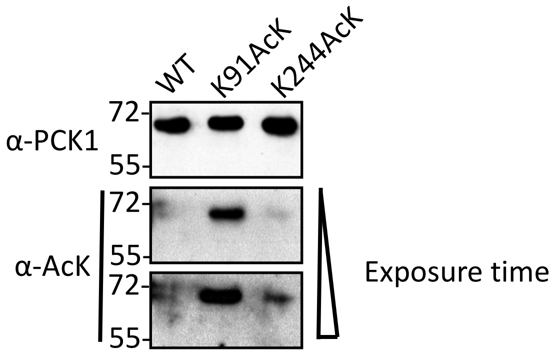

Figure S3. Anti-acetyl-lysine antibody (CST 9814) sensitivity to different acetyl-lysine targets. Two acetylated variants (K91AcK and K244AcK) were probed with the anti-acetyl-lysine antibody used in this work in order to determine possible differences in sensitivity.

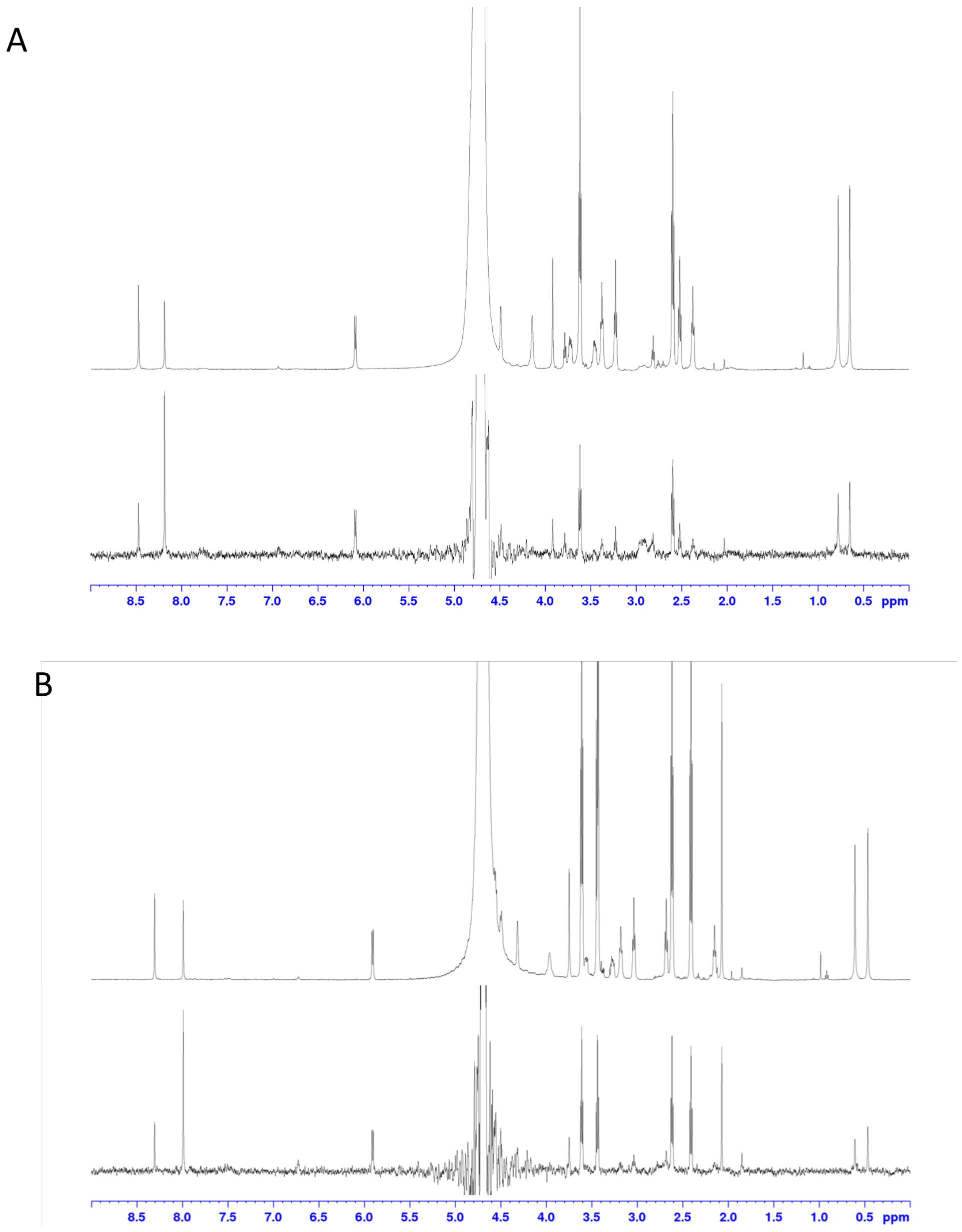

Figure S4. Representative STD NMR spectra (500 MHz) of (A) CoA and (B) Acetyl-CoA at 1 mM in the presence of 40 µM Pck1 at 283 K. In both cases, lower spectra correspond to the off-resonance experiment (reference) and the upper ones to the difference (STD) spectra.

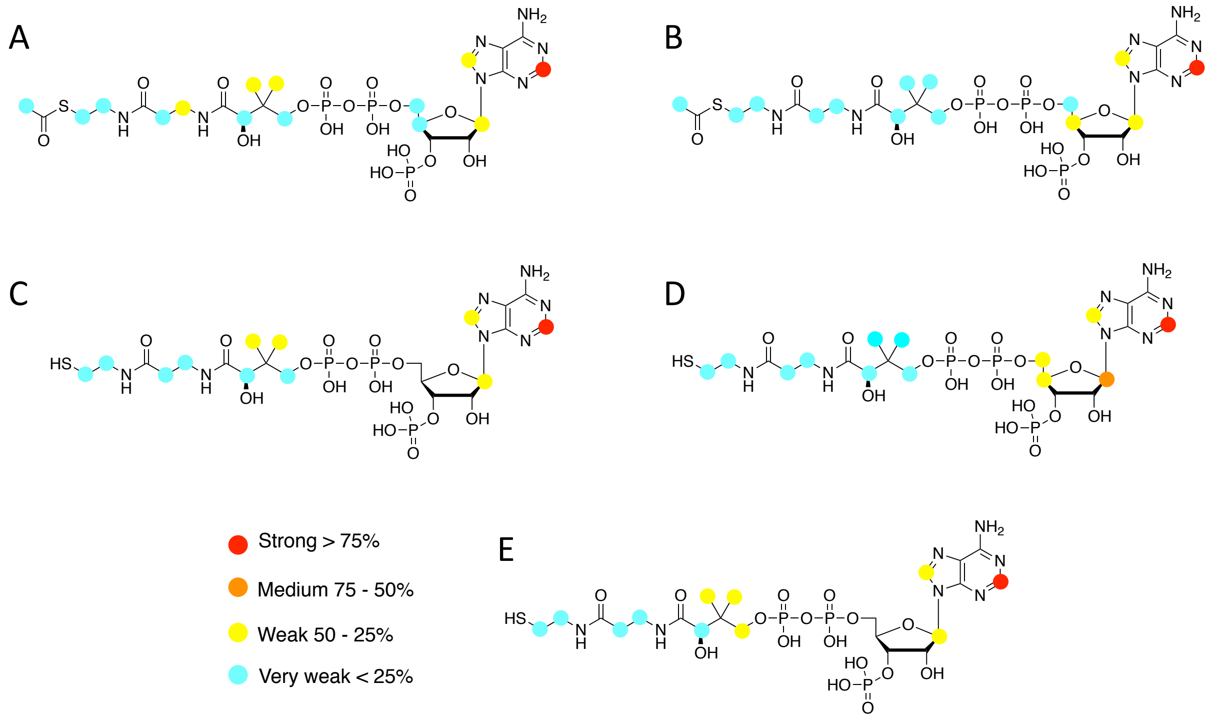

Figure S5. Mapping of ligand binding epitopes for their interactions with PCK1 by STD NMR. (A) AcCoA, saturation frequency -1 ppm. (B) AcCoA, saturation frequency 7.19 ppm. (C) CoA, saturation frequency - 1 ppm. (D) CoA, saturation frequency 7.19 ppm. (E) CoA, $MgCl_2$ 1 mM, saturation frequency - 1s ppm. The colored spheres represent the normalized STD-NMR intensity. Only STD responses are indicated for those protons that could accurately be measured.

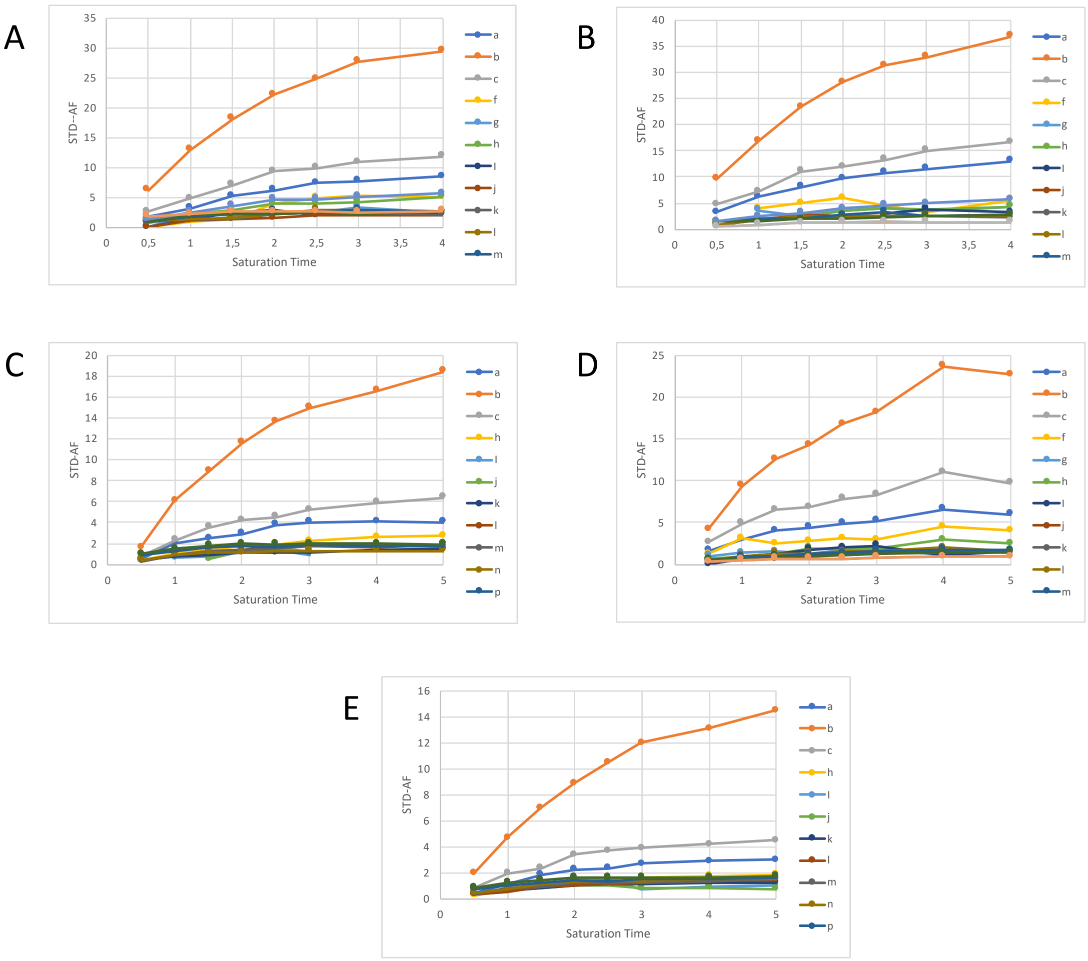

Figure S6. STD NMR build-up curves for each ligand in complex with PCK1, at different frequencies and in the presence of $MgCl_2$. (A) AcCoA, saturation frequency -1 ppm. (B) AcCoA, saturation frequency 7.19 ppm (C) CoA, saturation frequency - 1 ppm. (D) CoA, saturation frequency 7.19 ppm. (E) CoA, $MgCl_2$ 1 mM, saturation frequency - 1 ppm. Initial slopes were calculated and normalized to determine the epitope mappings.

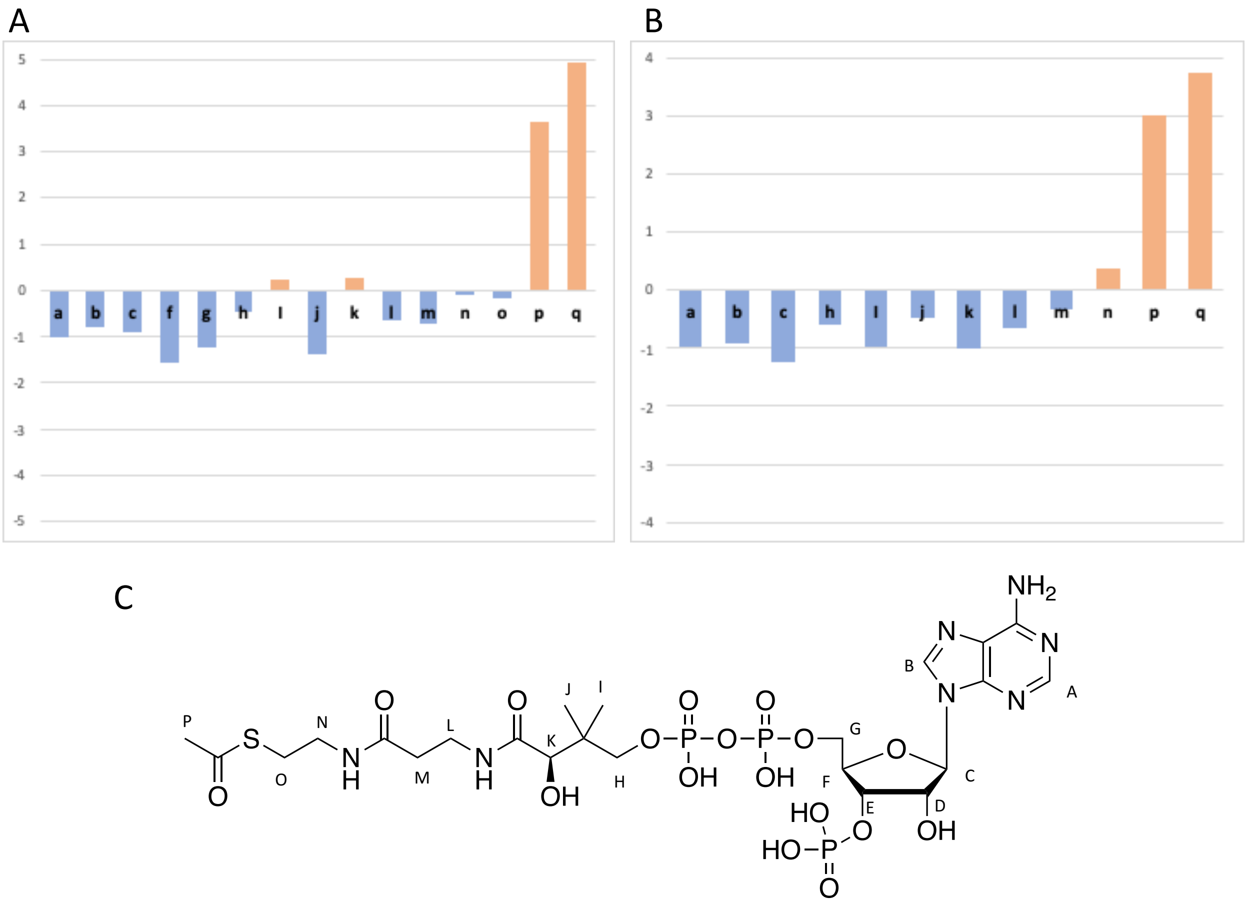

Figure S7. Differential Epitope Mapping histograms (-1/7.19 ppm) for each ligand in complex to PCK1. (A) AcCoa/PCK1 complex. (B) CoA/PCK1 complex. Positive ΔSTDs shown in orange indicate ligand protons oriented toward aliphatic protein side chains in the binding pocket (δ = -1 ppm) , and negative ΔSTDs, in blue, highlight ligand protons oriented toward aromatic side chains (δ = 7.19 ppm). ΔSTD values are calculated following described method. (C) Key with letter code.

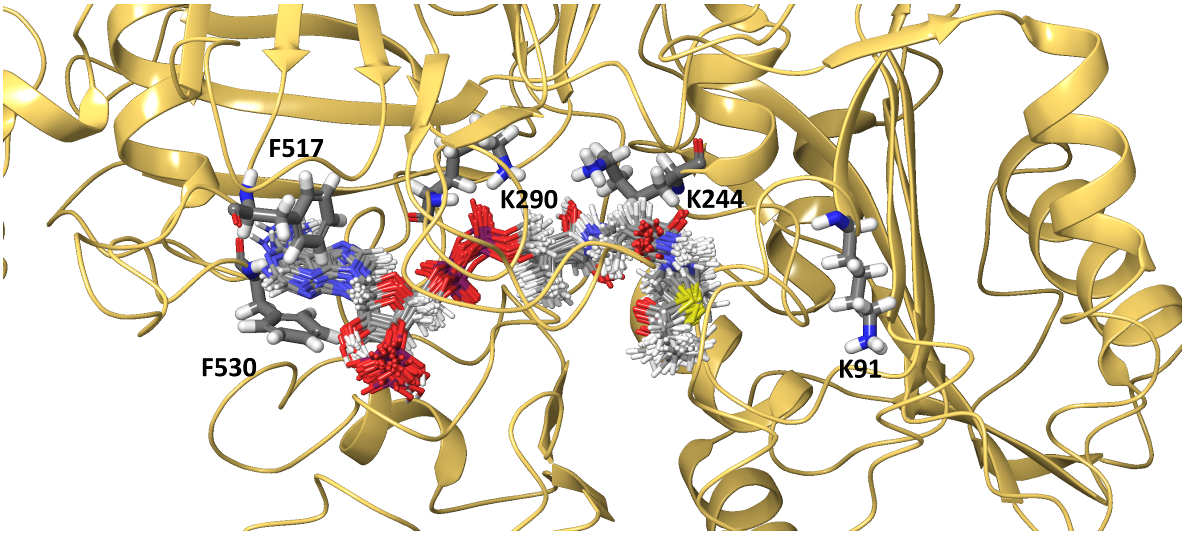

Figure S8. 3D molecular model of the Pck1-AcCoA complex. Superposition of 25 frames from a short 5ns MD simulation.

Table S1. Kinetic characterization of Pck1 WT and Pck1 WT incubated in the presence of sodium acetate (NaAc-treated) acetyl-CoA (AcCoA-treated) or acetyl CoA and p300 (p300-treated).

Substrate	Parameter	WT	NaAc-treated	AcCoA-treated	P300-treated
**A) OAA+GTP→ PEP+GDP+CO_2**					
**OAA**	K_m (µM)	45±9	45±8	65±16	81±16
	k_{cat} (s^{-1})	45±2	39±1.5	13±1	11±1
	k_{cat}/K_m ($M^{-1}s^{-1}$)	1 x 10^6	9 x 10^5	2 x 10^5	1.4 x 10^5
**GTP**	K_m (µM)	258±46	294±60	305±89	403±143
	k_{cat} (s^{-1})	41±3	44±3	7.3±0.9	9.5±1.5
	k_{cat}/K_m ($M^{-1}s^{-1}$)	1.6 x 10^5	1.5 x 10^5	2.4 x 10^4	2.4 x 10^4
**B) PEP+GDP+CO_2 → OAA+GTP**					
**PEP**	K_m (µM)	532±75	499±86	423±131	106±25
	k_{cat} (s^{-1})	19±1	20±1.3	6.3±0.6	8.1±0.5
	k_{cat}/K_m ($M^{-1}s^{-1}$)	3.6 x 10^4	4 x 10^4	1.5 x 10^4	7.6 x 10^4
**GDP**	K_m (µM)	87±10	61±14	64±11	50±9
	k_{cat} (s^{-1})	19±1	18±1.2	4.5±0.3	9.8±0.5
	k_{cat}/K_m ($M^{-1}s^{-1}$)	2.2 x 10^5	3 x 10^5	8.3 x 10^4	1.6 x 10^5
**$KHCO_3$**	K_m (mM)	14±2.2	12.6±1	8.7±2.7	11±2.6
	k_{cat} (s^{-1})	19±1	22±1.1	6±0.7	11±1
	k_{cat}/K_m ($M^{-1}s^{-1}$)	1.4 x 10^3	1.7 x 10^3	7 x 10^2	1.1 x 10^3

Table S2. Determination of lysine acetylation stoichiometry for PCK1 peptides (see supplementary spreadsheet)

Table S3. Data collection and refinement statistics. Values in parentheses refer to the highest resolution shell. Ramachandran plot statistics were determined with PROCHECK.

	PCK1-K244AcK
Space group	$P2_12_12_1$
Wavelength (Å)	0.97
Resolution (Å)	20-1.75<br>(1.85-1.75)
Cell dimensions<br>a, b, c (Å)<br>α, β, γ (°)	*a = 60.38*<br>*B* = 85.22<br>*c* = 119.67<br>*90, 90, 90*
Mn(I) half-set correlation CC(1/2)	0.996 (0.522)
Unique reflections	55039
Completeness	87.30 (99.8)
R_{pim}	0.043 (0.258)
$I/\sigma(I)$	13.2 (2.7)
Redundancy	5.7 (5.9)
R_{work} / R_{free}	0.170/0.211
RMSD from ideal geometry, bonds (Å)	0.0118
RMSD from ideal geometry, angles (º)	1.574
$\langle B \rangle$ PCK1-K244AcK (Å^2)	23.91
$\langle B \rangle$ solvent (Å^2)	33.44
PDB ID	6YI9
